# Supplementary material for: DNA Metabarcoding as a Tool for Disentangling Food Webs in Agroecosystems
Source: Insects. 2020 May 11;11(5):294. doi: 10.3390/insects11050294 (PMC7290477; doi:10.3390/insects11050294)
Supplement: Supplementary file 1 [file insects-11-00294-s001.zip › Table S1.docx]

|  |  | Arthropod sequencing run* | | | Vertebrate sequencing run | | |
| --- | --- | --- | --- | --- | --- | --- | --- |
| Filter step | **Objectifs** | **Nb of samples** | **Nb of OTUS** | **Nb of sequences** | **Nb of samples** | **Nb of OTUS** | **Nb of sequences** |
| Raw abundance table | NA | 1431 | 9116 | 7567854 | 685 | 3531 | 2671035 |
| Threshold TCC | Filter out cross-contaminations generated during the presequencing procedures using the maximal number of reads per variant observed in various negative controls | 1386 | 8997 | 7465638 | 663 | 3529 | 2661675 |
| Threshold TFA | Filter out misindexing generated during the sequencing using the rate of read false assignment calculated thanks to a DNA internal control | 1371 | 8832 | 7372315 | 654 | 3524 | 2618208 |
| PCR replicates | Eliminate inconsistent results between the three PCR replicates from the same sample to remove putative false-positive occurrences | 1362 | 516 | 7290353 | 540 | 1444 | 2016124 |

**Table S1.** Objectifs and impacts of the data filtering steps (TCC, TFA, PCR replicate) on the number of samples, OTUS, and sequences in the abundance table

*This run include arthropod predators analyzed in this study, and additional samples concerning other study.
